# PKC-mediated phosphorylation and activation of the MEK/ERK pathway as a mechanism of acquired trastuzumab resistance in HER2-positive breast cancer

**DOI:** 10.3389/fendo.2022.1010092

**Published:** 2022-10-18

**Authors:** Jeanesse Scerri, Christian Scerri, Felix Schäfer-Ruoff, Simon Fink, Markus Templin, Godfrey Grech

**Affiliations:** ^1^ Department of Physiology & Biochemistry, University of Malta, Msida, Malta; ^2^ NMI Natural and Medical Sciences Institute, University of Tübingen, Reutlingen, Germany; ^3^ Department of Pathology, University of Malta, Msida, Malta

**Keywords:** acquired resistance, breast cancer, phospho-profile, PKC/MEK/ERK, signalosome, HER2 positive, patient stratification

## Abstract

Protein expression, activation and stability are regulated through inter-connected signal transduction pathways resulting in specific cellular states. This study sought to differentiate between the complex mechanisms of intrinsic and acquired trastuzumab resistance, by quantifying changes in expression and activity of proteins (phospho-protein profile) in key signal transduction pathways, in breast cancer cellular models of trastuzumab resistance. To this effect, we utilized a multiplex, bead-based protein assay, DigiWest^®^, to measure around 100 proteins and protein modifications using specific antibodies. The main advantage of this methodology is the quantification of multiple analytes in one sample, utilising input volumes of a normal western blot. The intrinsically trastuzumab-resistant cell line JIMT-1 showed the largest number of concurrent resistance mechanisms, including PI3K/Akt and RAS/RAF/MEK/ERK activation, β catenin stabilization by inhibitory phosphorylation of GSK3β, cell cycle progression by Rb suppression, and CREB-mediated cell survival. MAPK (ERK) pathway activation was common to both intrinsic and acquired resistance cellular models. The overexpression of upstream RAS/RAF, however, was confined to JIMT 1; meanwhile, in a cellular model of acquired trastuzumab resistance generated in this study (T15), entry into the ERK pathway seemed to be mostly mediated by PKCα activation. This is a novel observation and merits further investigation that can lead to new therapeutic combinations in HER2-positive breast cancer with acquired therapeutic resistance.

## Introduction

### HER2 and trastuzumab

The human epidermal growth factor receptor 2 (HER2) protein is overexpressed in approximately 15% of breast cancers (1). Having no known ligands, it forms heterodimers with other members of the HER family of receptor tyrosine kinases (HER1/EGFR, HER3, HER4 (2). HER2 activation results in the phosphorylation and activation of multiple downstream signaling proteins, including phospholipase C γ1 (PLCγ1), phosphatidylinositol 3-kinase (PI3K) regulatory and catalytic subunits, RasGAP, and heat shock protein 90 (3). The ensuing signaling cascade, mostly represented by the PI3K/AKT and RAS/RAF/ERK pathways, leads to uncontrolled cellular proliferation and invasion. Protein phosphatase 2A (PP2A), a ubiquitous serine/threonine phosphatase, is also a central regulatory component of PI3K/Akt pathway; its inactivation through phosphorylation at its tyrosine residue p.tyr307 has been found to be increased in HER2-positive tumor samples and correlated to tumor progression (4). Of interest, HER2 signaling increases c-myc phosphorylation at Ser62 and is maintained through attenuation of the phosphatase, PP2A (5). In fact, PP2A activators promote c-myc protein degradation (6). Clinically, high nuclear myc staining is positively associated with lymph-node positive disease in HER2 amplified breast cancer tumors (7). Hence, the HER2-MYC-PP2A axis is of clinical relevance and provides potential therapeutic targeting of breast cancers with co-amplification of HER2 and MYC. In a murine model of HER2 knock-in mammary tumors, overexpression of HER2 significantly upregulated β-catenin and its transcriptional targets Cyclin D1, SOX9 and c-Myc. High cytoplasmic β-catenin, expression of basal markers and loss of membranous E-cadherin are associated with poor prognosis in human HER2+ invasive ductal carcinomas (8).

Trastuzumab (Herceptin^®^), an immunoglobulin G1 (IgG1) antibody consisting of two mouse-derived antigen binding sites specific to the HER2 receptor extracellular domain (ED) and a humanized Fc portion (9), has been hailed as one of the successes of personalized medicine for the treatment of HER2-positive breast cancer. Its mode of action, though not yet fully understood, involves both direct and indirect pathways of inhibition. The former is brought about by the binding of the antibody to the ED of Her2, inhibiting its cleavage (10), and resulting in downstream signaling inhibition (mainly the PI3K/Akt pathway (11), through internalization and degradation of the HER2 receptor (12). The inhibition of heterodimer formation with other HER family members leads to reduced VEGF-mediated angiogenesis (13). The most important indirect pathway of inhibition is the activation of antibody-dependent cellular toxicity by the recruitment of Fc-competent immune effector cells (14). Trastuzumab is always administered adjuvantly to chemotherapeutic agents, where it also inhibits the repair of chemotherapy-induced DNA damage (15).

### Trastuzumab resistance mechanisms

Nonetheless, intrinsic resistance to the drug in some cases, and tumour recurrence due to acquired resistance in others, are important caveats of the targeted therapy (16). Mechanisms of trastuzamab-HER2 binding inhibition are associated with intrinsic resistance. Steric hindrance by cell surface proteins such as mucin-4 (MUC4) inhibits this binding (17); sensitivity to trastuzumab was enhanced upon knockdown of MUC4 expression in a JIMT-1 cell model (18), suggesting that MUC4 occupies the trastuzamab-binding sites of HER2. Overexpression of stem cell marker CD44 and its ligand, hyaluronan, also mask the trastuzumab binding domain on the HER2 ED and provide an independent prognostic factor for poor disease-free survival in HER2 positive patients treated with adjuvant trastuzumab (19). Proteolytic cleavage of the HER2 receptor generates a constitutively activated, truncated HER2 receptor lacking the ED, p95-HER2, which is associated with lymph node involvement (20) and trastuzumab resistance (21), attributed to the absence of the trastuzumab-binding domain.

Deregulation of signalling pathways downstream to HER2, and the activation of alternative cellular proliferation pathways, are alternative trastuzumab resistance mechanisms. Suppressed PTEN phosphatase activity prevents trastuzumab-induced growth arrest through sustained PI3K/AKT phosphorylation and signal transduction (22). A combination of low PTEN expression and PIK3CA oncogenic mutations predict trastuzumab response in HER2-positive breast cancer patients (23). In addition, trastuzumab-induced growth arrest of HER2-positive tumour cells is counteracted by an increase in insulin-like growth factor-1 receptor (IGF-IR) signalling (24). IGF-IR mediated trastuzumab-resistance is attributed to enhanced degradation of p27 and hence release from cell cycle arrest induced by trastuzumab treatment (25). Resistance to trastuzumab was also associated with increased expression of c-Met (26), and CAV-1 involved in caveolae-mediated endocytosis (27).

Immune escape is another mechanism of trastuzumab resistance. Genomic polymorphisms in FcγRIIIa that significantly suppress the affinity of IgG1 antibodies to the immune cell Fcγ receptor will impair ADCC activation (28). Furthermore, exosomes may transfer transforming growth factor beta 1 (TGFβ1), an immunosuppressive cytokine, and programmed death-ligand-1 (PD-L1), a lymphocyte activation inhibitor, to tumour cells. The presence of these exosomes was correlated with resistance to ADCC, suggesting a role of exosomes in suppressed immune-mediated response to trastuzumab (29). Exosomes generated by SKBR3 cell lines are also positive for the receptor, and may act as decoy by binding to trastuzumab, reducing its availability to target tumour cells (30).

### High-throughput biomarker detection

In addition to diagnostic biomarkers, the discovery of predictive markers of treatment resistance is a key aspect of personalized medicine. In the era of network medicine and high-throughput “omics”, it is important to study the interplay of the different complex mechanisms leading to drug resistance. The classification of breast cancer into molecular subtypes with prognostic and predictive implications, based on high-throughput gene expression data, has led to the development of gene panels such as the Oncotype DX (31) or the MammaPrint™ (32) assays. For Her2-positive breast cancer, however, there is no FDA-approved gene panel to date for the clinical prediction of response to trastuzumab-containing treatment regimes. The use of bead-based, multiplex RNA (33) and protein (34) assays has shown effectiveness in medium- to high-throughput cancer biomarker discovery and detection.

This study sought to differentiate between the complex mechanisms of intrinsic and acquired trastuzumab resistance, by quantifying changes in expression and activity of proteins in key signal transduction pathways, in cellular models of resistance. We utilized JIMT-1 as a cellular model of intrinsic resistance, and generated an acquired trastuzumab resistance model (T15) to study differential signaling signatures.

## Materials and methods

### Generation of trastuzumab-resistant cell line

SKBR3 cells with acquired trastuzumab resistance were obtained by conditioning with the drug as described by Zazo et al. (35). Briefly, the cell line (ATCC^®^ HTB-30™), grown in Dulbecco’s Modified Eagle Medium (DMEMM, Sigma-Aldrich, St. Louis, MO) supplemented with 10% foetal bovine serum (FBS) and 1% GlutaMAX™ (Thermo Fisher Scientific, Waltham, MA), was acclimatised for 30 days in 10µg/mL trastuzumab followed by long-term culturing in medium containing 15µg/mL of the drug. Resistance to trastuzumab was confirmed by cell viability assay (MTT), which showed a maintenance of ≥80% viability after 72 hours incubation with 25-100µg/mL trastuzumab concentration (compared to the parent cells which showed reduced viability at these drug concentrations). The resulting cell line will be henceforth referred to as T15. The JIMT-1 cell line (DSMZ ACC-589), kindly donated by M. Barok at the University of Helsinki, Finland, was cultured in DMEM supplemented with 10% heat-inactivated FBS.

### Bead-based, multiplex phosphoprotein profiling

High-throughput multiplex phosphoprotein profiling was subsequently carried out by the DigiWest^®^ technique, as described by Treindl et al. (36), on the parental and conditioned cell lines. Briefly, cell pellets containing 5x10^5^ cells or more were lysed, and gel electrophoresis and blotting onto PVDF membranes was performed using the NuPAGE system as recommended by the manufacturer (Life Technologies, Carlsbad, CA, USA). The membranes were washed in PBST, then incubated in NHS-PEG12-Biotin (50μM) in PBST for 1 hour to biotinylate the blotted proteins, followed by another wash in PBST and drying. Individual sample lanes were cut into 96 molecular weight fractions (0.5mm each), with the separated proteins in each fraction eluted in 96-well plates using 10μL elution buffer (8M urea, 1% Triton-X100 in 100mM Tris-HCl pH 9.5) per well. The eluted proteins from each molecular weight fraction were then coupled with neutravidin-coated Luminex beads (MagPlex, Luminex, Austin, TX, USA) of a specific bead identity (red-infrared spectral wavelength), yielding 96 size-specific bead identities per sample. 384 Luminex bead sets were employed and the protein-loaded beads from 4 different sample lanes were pooled into a bead-mix having a concentration of 40 beads/µL in carboxy block storage buffer (CBS), which was sufficient for over 100 antibody incubations. Antibodies specific proteins and phosphoproteins with roles in HER2 downstream signaling pathways and other aforementioned mechanisms of interest were utilized (**Table 1**).

**Table 1:** Selected antibodies, fluorescence intensities and Log_2_ FC in protein & phosphoprotein quantities in T15 and JIMT-1 relative to SKBR3.

					Fluorescence Intensity			LOG2 FC rel. to SKBR3	
Pathway	Analyte	Supplier	Cat. No.	Species + Clonality	JIMT1	SKBR3	T15	JIMT1	T15
PI3K/mTOR	4E-BP1	Epitomics	1557-1	Rb mAb	2490	240	283	3.37	0.24
PI3K/mTOR	4E-BP1 - phosphoThr70	Cell Signaling	9455	Rb pAb	754	119	164	2.66	0.46
PI3K	Akt	Cell Signaling	4685	Rb mAb	3567	2125	2901	0.75	0.45
PI3K	Akt1	Cell Signaling	2938	Rb mAb	1105	1443	2341	-0.39	0.70
PI3K	Akt1 - phosphoSer129	Cell Signaling	13461	Rb mAb	1	1	328	0.00	8.36
mTOR	AMPK alpha	Cell Signaling	2532	Rb pAb	657	418	544	0.65	0.38
mTOR	AMPK alpha - phosphoThr172	Cell Signaling	2535	Rb mAb	1217	135	300	3.17	1.15
MEK/ERK	A-Raf	Cell Signaling	4432	Rb pAb	2952	1177	1051	1.33	-0.16
MEK/ERK	A-Raf - phosphoTyr301/Tyr302_58kDa	Biorbyt	orb5910	Rb pAb	28345	31881	29893	-0.17	-0.09
MEK/ERK	A-Raf - phosphoTyr301/Tyr302_68kDa	Biorbyt	orb5910	Rb pAb	21540	22850	19923	-0.09	-0.20
MEK/ERK	A-Raf - phosphoTyr301/Tyr302_Total	Biorbyt	orb5910	Rb pAb	49885	54729	49815	-0.13	-0.14
PI3K/WNT	beta-Catenin	Cell Signaling	8480	Rb mAb	24084	188	382	7.00	1.02
PI3K/WNT	beta-Catenin - phosphoSer552	Cell Signaling	9566	Rb pAb	449	1	1	8.81	0.00
PI3K/WNT	beta-Catenin (non-pospho Ser33/37/Thr41; active)	Cell Signaling	8814	Rb mAb	3541	1	1	11.79	0.00
MEK/ERK	b-Raf - phosphoSer445	Cell Signaling	2696	Rb pAb	208	143	160	0.54	0.16
Cell cycle	CDK4	Cell Signaling	12790	Rb mAb	32451	3328	2931	3.29	-0.18
PI3K	c-myc_57kDa	Cell Signaling	9402	Rb pAb	207	205	241	0.01	0.23
PI3K	c-myc_70kDa	Cell Signaling	9402	Rb pAb	549	520	337	0.08	-0.63
PI3K	c-myc_Total	Cell Signaling	9402	Rb pAb	756	724	577	0.06	-0.33
MEK/ERK	c-Raf	Cell Signaling	9422	Rb pAb	632	161	135	1.97	-0.25
MEK/ERK	c-Raf - phosphoSer259	Cell Signaling	9421	Rb pAb	2423	873	874	1.47	0.00
MEK/ERK	c-Raf - phosphoSer289/296/301	Cell Signaling	9431	Rb pAb	374	186	168	1.01	-0.15
PI3K	CREB - phosphoSer133	Cell Signaling	9198	Rb mAb	177	1	56	7.47	5.80
PI3K	eIF4E	Cell Signaling	2067	Rb mAb	13776	16186	17424	-0.23	0.11
PI3K	eIF4E - phosphoSer209	Cell Signaling	9741	Rb pAb	348	927	1193	-1.41	0.36
MEK/ERK	Elk-1	Cell Signaling	9182	Rb pAb	653	656	813	-0.01	0.31
MEK/ERK	Elk-1 - phosphoSer383	Cell Signaling	9186	ms mab	644	1580	1651	-1.29	0.06
MEK/ERK	Erk1/2 (MAPK p44/42)_p42	Cell Signaling	4695	Rb mAb	17917	30972	41263	-0.79	0.41
MEK/ERK	Erk1/2 (MAPK p44/42)_p44	Cell Signaling	4695	Rb mAb	3100	1702	1774	0.87	0.06
MEK/ERK	Erk1/2 (MAPK p44/42)_Total	Cell Signaling	4695	Rb mAb	21016	32673	43036	-0.64	0.40
MEK/ERK	Erk1/2 (MAPK p44/42) - phosphoThr202/Tyr204_p42	Cell Signaling	4370	Rb mAb	4211	788	1190	2.42	0.60
MEK/ERK	Erk1/2 (MAPK p44/42) - phosphoThr202/Tyr204_p44	Cell Signaling	4370	Rb mAb	1656	93	294	4.16	1.67
MEK/ERK	Erk1/2 (MAPK p44/42) - phosphoThr202/Tyr204_Total	Cell Signaling	4370	Rb mAb	5866	880	1482	2.74	0.75
MEK/ERK	ERK1/2 (MAPK) - phosphoThr202/Tyr204_p42	Cell Signaling	9101	Rb pAb	4653	257	480	4.18	0.90
MEK/ERK	ERK1/2 (MAPK) - phosphoThr202/Tyr204_p44	Cell Signaling	9102	Rb pAb	1340	113	143	3.57	0.34
MEK/ERK	ERK1/2 (MAPK) - phosphoThr202/Tyr204_Total	Cell Signaling	9103	Rb pAb	5993	368	621	4.02	0.75
MEK/ERK	Erk2 (MAPK p42)	Cell Signaling	9108	Rb pAb	2649	6720	10792	-1.34	0.68
WNT	GSK-3 alpha	Cell Signaling	4337	Rb mAb	4395	4403	4928	0.00	0.16
WNT	GSK3 alpha - phosphoSer21_51kDa	Cell Signaling	9331	Rb pAb	229	443	409	-0.95	-0.11
PI3K/WNT	GSK3 alpha/beta - phosphoSer21/Ser9_Total	Cell Signaling	9331	Rb pAb	548	443	409	0.31	-0.11
PI3K	GSK3 beta - phosphoTyr216_47kDa	Abcam	ab68476	Rb mAb	129	232	281	-0.85	0.28
PI3K	GSK3 alpha - phosphoTyr279_51kDa	Abcam	ab68476	Rb mAb	706	125	148	2.50	0.25
PI3K	GSK3 alpha/beta - phosphoTyr279/Tyr216_Total	Abcam	ab68476	Rb mAb	834	355	429	1.23	0.27
PI3K	GSK3 beta - phosphoSer9	Cell Signaling	9336	Rb pAb	511	1	1	9.00	0.00
PI3K	GSK3 beta	Cell Signaling	9315	Rb mAb	11035	3642	2050	1.60	-0.83
HER2	Her2	DAKO	A0485	Rb pAb	3595	5008	8862	-0.48	0.82
Multiple	HSP 90	Abcam	ab59459	Ms mAb	150805	456009	861935	-1.60	0.92
IGF1	IGF1 receptor beta (Insulin receptor beta, CD221)	Cell Signaling	3018	Rb mAb	308	153	166	1.02	0.12
MEK/ERK	MAPKAPK-2	Cell Signaling	12155	Rb mAb	392	556	453	-0.50	-0.29
MEK/ERK	MEK 1	Cell Signaling	9124	Rb pAb	1128	610	644	0.89	0.08
MEK/ERK	MEK1 - phosphoSer298	Cell Signaling	98195	Rb mAb	896	1	1	9.81	0.00
MEK/ERK	MEK1 - phosphoThr292	Cell Signaling	26975	Rb mAb	1036	1	1	10.02	0.00
MEK/ERK	MEK1/2 - phosphoSer217/Ser221	Cell Signaling	9154	Rb mAb	3200	135	416	4.56	1.62
MEK/ERK	MEK2	Cell Signaling	9125	Rb pAb	953	171	131	2.48	-0.38
MEK/ERK	Mnk1	Cell Signaling	2195	Rb mAb	239	161	164	0.57	0.03
MEK/ERK	MSK1 - phosphoSer376	Millipore	04-384	Rb mAb	2302	2436	9259	-0.08	1.93
PI3K/mTOR	mTOR (FRAP)	Cell Signaling	2983	Rb mAb	3077	1394	2232	1.14	0.68
PI3K/mTOR	mTor - phosphoSer2448	Cell Signaling	5536	Rb mAb	1211	521	997	1.22	0.94
MEK/ERK	p38 MAPK	Cell Signaling	9212	Rb pAb	572	258	273	1.15	0.08
Cell cycle	p53	R&D	af1355	Gt pAb	9935	1601	2117	2.63	0.40
PI3K/mTOR	p70 S6 kinase	Cell Signaling	2708	Rb mAb	5905	2365	3004	1.32	0.34
PI3K/mTOR	p70 S6 kinase - phosphoThr421/Ser424	Cell Signaling	9204	Rb pAb	632	93	177	2.76	0.92
PI3K	PDK1	Cell Signaling	3062	Rb pAb	1398	808	1294	0.79	0.68
PI3K	PDK1 - phosphoSer241	Cell Signaling	3061	Rb pAb	142	73	193	0.96	1.39
PI3K	PI3-kinase p110 delta_110kDa	Santa cruz	sc-7176	Rb pAb	734	220	238	1.74	0.11
PI3K	PI3-kinase delta_60kDa	Santa cruz	sc-7176	Rb pAb	11463	12042	11384	-0.07	-0.08
PI3K	PI3-kinase delta_Total	Santa cruz	sc-7176	Rb pAb	12196	12262	11620	-0.01	-0.08
PI3K	PI3-kinase p110 alpha	Cell Signaling	4255	Rb pAb	31	253	266	-3.05	0.07
PI3K	PI3-kinase p110 beta_110kDa	Millipore	04-400	Rb mAb	2897	916	995	1.66	0.12
PI3K	PI3-kinase p110 beta_60kDa	Millipore	04-400	Rb mAb	1514	1835	1665	-0.28	-0.14
PI3K	PI3-kinase p110 beta_Total	Millipore	04-400	Rb mAb	4409	2750	2659	0.68	-0.05
PI3K	PI3-kinase p85 alpha	Epitomics	1675-1	Rb mAb	363	87	118	2.06	0.44
PI3K	PI3-kinase p85	Cell Signaling	4292	Rb pAb	437	129	157	1.76	0.28
PI3K	PI3-kinase p85/p55 - phosphoTyr458/Tyr199_55kDa only	Cell Signaling	4228	Rb pAb	336	3158	3154	-3.23	0.00
PI3K	PKC (pan) - phosphoSer660	Cell Signaling	9371	Rb pAb	1073	1180	4669	-0.14	1.98
PI3K	PKC (pan) gamma - phosphoThr514_80kDa	Cell Signaling	38938	Rb mAb	1102	1141	2533	-0.05	1.15
PI3K	PKC (pan) gamma - phosphoThr514_85kDa	Cell Signaling	38938	Rb mAb	2613	3150	5891	-0.27	0.90
PI3K	PKC (pan) gamma - phosphoThr514_Total	Cell Signaling	38938	Rb mAb	3715	4290	8423	-0.21	0.97
PI3K	PKC alpha - phosphoSer657	Abcam	AB180848	Rb mAb	1769	1122	4227	0.66	1.91
PI3K	PKC alpha - phosphoThr497	Abcam	AB76016	Rb mAb	1526	1948	2841	-0.35	0.54
PI3K	PKC alpha	BD Biosciences	610107	Ms mAb	1	77	80	-6.27	0.05
PI3K	PKC alpha/beta II - phosphoThr638/Thr641	Cell Signaling	9375	Rb pAb	980	1570	1473	-0.68	-0.09
PI3K	PP2A C	Cell Signaling	2259	Rb mAb	5653	3171	2571	0.83	-0.30
PI3K	PP2A C - phosphoTyr307	R&D	AF3989	Rb pAb	4986	20990	10084	-2.07	-1.06
PI3K	PTEN	Cell Signaling	9552	Rb pAb	228	203	304	0.16	0.58
MEK/ERK	Ras	Cell Signaling	8955	Rb mAb	2280	742	1103	1.62	0.57
Cell cycle	Rb	Cell Signaling	9309	Ms mAb	405	131	119	1.62	-0.15
Cell cycle	Rb - phosphoSer795	Cell Signaling	9301	Rb pAb	149	53	1	1.49	-5.73
Cell cycle	Rb - phosphoSer807/Ser811	Epitomics	2004-1	Rb mAb	1618	342	347	2.24	0.02
Multiple	RSK 1 (p90RSK)	Cell Signaling	9344	Rb pAb	1038	306	334	1.76	0.13
Multiple	RSK 1 (p90RSK) - phosphoSer380	Cell Signaling	9341	Rb pAb	317	228	853	0.48	1.90
Multiple	RSK 1 (p90RSK) - phosphoThr573	Abcam	ab62324	Rb mAb	556	115	303	2.27	1.40
Multiple	RSK 1/2/3	Cell Signaling	9347	Rb pAb	1059	438	453	1.27	0.05
Multiple	RSK 3	Epitomics	2012-1	Rb mAb	1005	409	648	1.30	0.67
Multiple	RSK 3 - phosphoThr356/Ser360	Cell Signaling	9348	Rb pAb	87	1	104	6.45	6.70
PI3K/mTOR	S6 ribosomal protein	Cell Signaling	2317	Ms mAb	6810	14092	11270	-1.05	-0.32
PI3K/mTOR	S6 ribosomal protein - phosphoSer235/Ser236	Cell Signaling	2211	Rb pAb	11823	31835	20663	-1.43	-0.62
PI3K/mTOR	S6 ribosomal protein - phosphoSer240/Ser244	Cell Signaling	2215	Rb pAb	9573	38584	21969	-2.01	-0.81
PI3K/mTOR	TSC2 (Tuberin)	Cell Signaling	4308	Rb mAb	2138	489	1073	2.13	1.13
PI3K/mTOR	Tuberin/TSC2 - phosphoSer1387	Cell Signaling	23402	Rb mAb	1369	233	564	2.55	1.27

Antibodies were organized into the main canonical pathways of signal transduction and cellular proliferation. Antibody species: Rb: rabbit, Ms: mouse, Gt: goat; antibody clonality: mAb: monoclonal, pAb: polyclonal. Fluorescence intensity values less than 100 are deemed inaccurate and should be interpreted with caution. Fold changes ≥1 are denoted in light orange and fold changes ≤ -1 are denoted in light green.

For each target protein or phosphoprotein to be quantified, an aliquot of the DigiWest bead-mixes was added to a well of a 96-well plate containing 50μL assay buffer (Blocking Reagent for ELISA supplemented with 0.2% milk powder, 0.05% Tween-20, and 0.02% sodium azide, Roche). Following a brief incubation in assay buffer, the buffer was discarded by keeping the 96-well plate on a magnet. The beads were then incubated with 30µL of a specific primary antibody diluted in assay buffer per well. After overnight incubation at 15°C on a shaker, the bead-mixes were washed twice with PBST and PE-labelled (Phycoerythrin) secondary antibodies (Dianova) specific to the primary antibody species were added and incubated for 1 hour at 23°C. Beads were washed twice and resuspended in PBST prior to the readout on a Luminex^®^ FlexMAP 3D^®^.

For the quantification of the antibody specific signals, the DigiWest^®^ analysis tool (version 3.8.6.1, Excel-based) was employed. This tool uses the 96 values for each initial lane obtained from the Luminex^®^ measurements on the 96 molecular weight fractions, identifies the peaks at the appropriate molecular weight, calculates a baseline using the local background, and integrates the peaks. The obtained values are based on relative fluorescence (AFI, accumulated fluorescence intensity). For analysis, the data was normalized to the total protein amount corresponding to the sample, and the relative quantification of each protein and phosphoprotein was expressed as log_2_ fold-change (FC) in T15 and JIMT-1 as compared to SKBR3. Differentially expressed targets were organized into established signal transduction pathways and phosphosite log_2_ FC were used to predict whether each protein was under- or over-activated.

## Results and discussion

### MEK/ERK pathway is a central mechanism of acquired trastuzumab resistance

**Figure 1:**
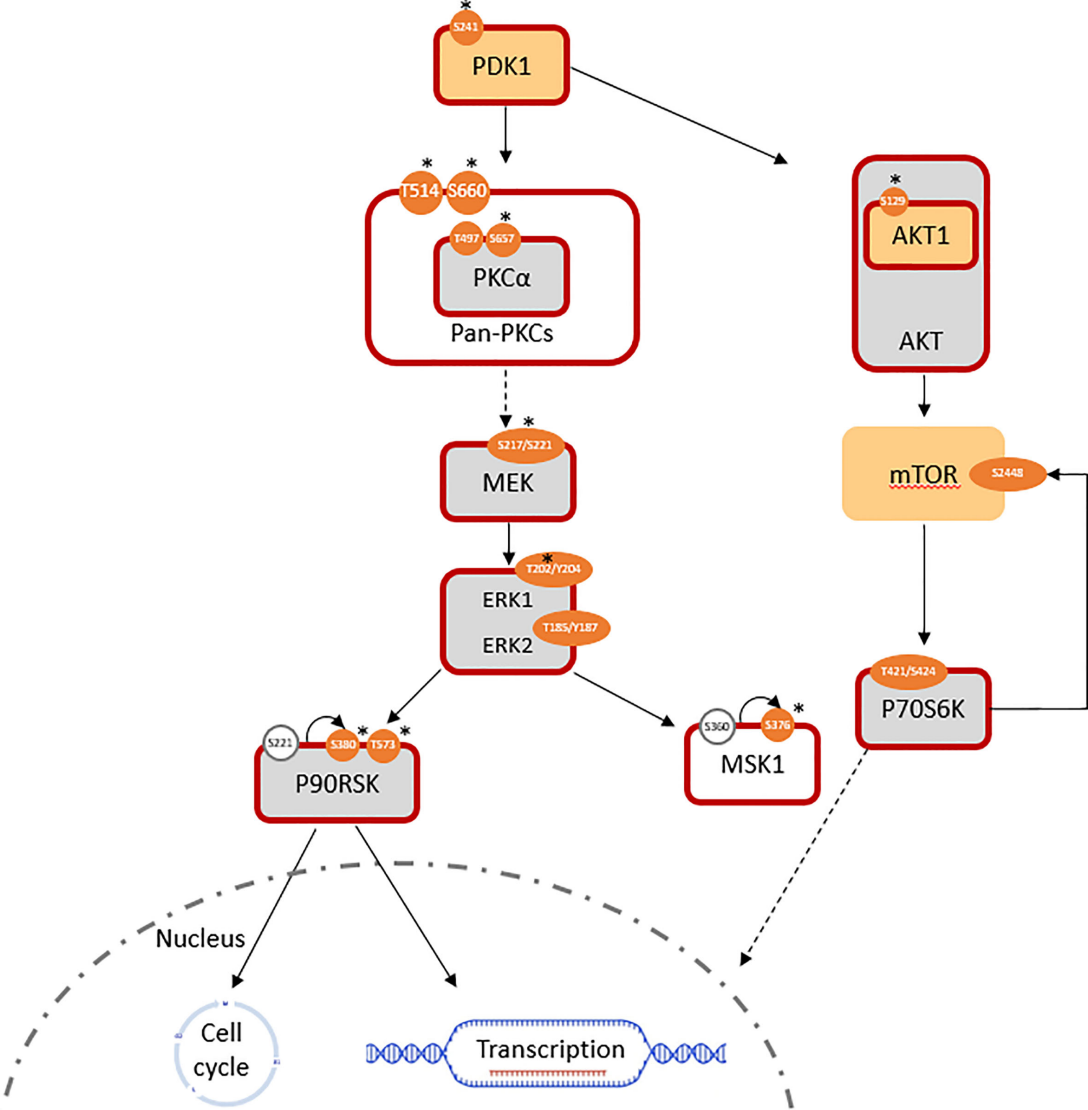
Pathways involved in the induced trastuzumab resistance of T15 (log_2_ FC normalized to SKBR3 signals). Similarly expressed protein: light grey; antibody not available/poor performance: no fill; overexpressed total protein ≥ 1.5-fold (log_2_ FC ≥ 0.58): light orange; ≥ 2-fold: *; over-represented phosphosite ≥ 1.5-fold: orange; ≥ 2-fold: *; predicted active protein: dark red outline.

Phosphoinositide-dependent kinase-1 (PDK1) activity was significantly increased (log_2_ FC(PDK1) = +0.7; log_2_ FC(pPDK1^ser241^ = +1.4) in T15. A lack of significant change in RAF expression was expected to be consistent with a lack of alteration in downstream MEK1/2 signaling; however, the MEK/ERK pathway was still found to be overall activated. The expression of total MEK1 was equivalent, while that of MEK2 was slightly downregulated (log_2_ FC = -0.36) in T15 when compared to SKBR3. Meanwhile, activated pMEK1^ser217/221^/pMEK2^ser222/226^ (antibody does not distinguish between the two isoforms) was significantly upregulated in T15 (log_2_ FC = +1.6). ERK (MAPK) activity reflected the changes observed in its upstream activator, MEK: despite minimal changes in total protein expression (log_2_ FC(ERK1) = +0.06, log_2_ FC(ERK2) = +0.41), phosphorylated (active) forms of ERK1 and ERK2 were over-represented, thus resulting in a higher ratio of phosphorylated to total ERK1/2 (log_2_ FC(pERK1^thr202/tyr204^ = +1.7; log_2_ FC(pERK2^thr185/tyr187^ = +0.6). The results were confirmed with two different antibody clones (Cell Signaling product ID 4370 and 9101; log_2_ fold changes reported here obtained with the former), both of which bind to ERK1 and ERK2 and give two specific peaks of 44 and 42 kDa, respectively (**Figure 1**; **Table 1** **)**. T15 also showed hyper-activation of the ribosomal protein S6 kinase α-5 protein, MSK1 (log_2_ FC(pMSK1^ser376^) = +1.9; **Figure 1**). MSK1 is directly phosphorylated by MAPKs at serine 360, threonine 581, and threonine 700, and subsequently autophosphorylates at serine 376 for protein activation (37). Seemingly conflicting roles for MSK1 in breast cancer have been described: it shows tumor suppressor functions by acting as a transcriptional coactivator of P53 and mediating phosphorylation of histone H3 in the transcriptional activation of *p21* (37), but has also been associated with epithelial-mesenchymal transition (EMT) and subsequent skeletal metastasis by histone H3 acetylation and phosphorylation of Snail, which downregulates E-cadherin to promote cellular migration and invasion (38).

### Activation of the MEK/ERK pathway is through PKCα activation in acquired resistance

In the absence of RAF overexpression, entry into the MEK/ERK pathway can be mediated by the protein kinase C (PKC) family, *via* PDK1. PKCα and PKCγ are both members of the diacylglycerol (DAG) sensitive, Ca^2+^ responsive conventional PKC (cPKC) isoform subgroup. Activation downstream to receptor tyrosine kinases, such as ErbB receptors, involves the Ca^2+^ sensitive recruitment of phosphatidylinositol (4, 5-bisphosphate [PtdIns (4, 5)P2]-specific phospholipases Cγ1/2 (PtdIns-PLCγ1/2) through their SH2 domains; PDK1-dependent activation loop phosphorylation, together with C-terminal phosphorylations events, catalyze PKC activity by maintaining the active conformation of the kinase domains (39). While PKCγ is more specific to the brain, PKCα is detected in all normal and most tumor tissue types (40). The presence of activated pan-PKC and specifically PKCα was determined by the over-representation of phospho-proteins in T15 (log_2_ FC(PKCA) = +0.05; log_2_ FC(pPKCA^thr497^) = +0.54; log_2_ FC(pPKCA^ser657^) = +1.91), as well as the overexpression of PDK-1 p-ser241, an autophosphorylation site essential for PDK1 activity (**Figure 1**). Increased levels of this phosphoprotein are a frequent event in breast cancer metastasis, and have been proposed as a candidate for chemosensitisation in innate and acquired resistance (41).

PKC-α, like other protein kinases, plays a role in the regulation of various cellular functions, ranging from cell proliferation and differentiation to control of apoptosis. Requiring HSP90 (log_2_ FC in T15 = 0.92) and mTORC2 complex to prime phosphorylation, it is sequentially phosphorylated at Thr497 in the kinase domain by PDK1 and at Thr638 and Ser657 autophosphorylation sites. While in the cytoplasm, the phosphorylated PKC-α is still inactive, until it is recruited to the plasma membrane, where it exerts its functions (42). Its importance in cellular proliferation renders its abnormal expression a transformative event: initial recognition of the role of PKC-α in tumorigenesis was reported by Ways and colleagues (43), where ectopic expression of the isoform in MCF7 cells led to a more aggressive phenotype characterized by increased cell proliferation, anchorage-independent growth, loss of epithelial morphology, and enhanced tumorigenicity in nude mice. Using the same cell line, Gupta et al. (44) attributed the increase in cellular proliferation to ERK activation by PKC-α.

PKC family members were also identified as kinases involved in HER2 endocytosis by Bailey and colleagues (45), by using tanespimycin to inactivate HSP90 (and thus promote receptor internalization for degradation), followed by a kinase inhibitor screen to identify kinases whose inhibition correlated with reduced cell surface clearance of HER2. The activation of PKC by phorbol myristate acetate (PMA), and the specific ectopic expression of constitutively active PKC-α, promoted its co-localization with HER2 into a juxtanuclear compartment without subsequent degradation. Conversely, knockdown of PKC-α by siRNA impaired HER2 trafficking to the ERC. In a previous study, PKC-α was implicated in the positive regulation of cell surface HER2 receptor levels, as assessed by flow cytometry, in breast cancer cell lines classified as HER2 2+ on immunohistochemistry without gene amplification as determined by fluorescence *in situ* hybridization (FISH) (46).

### Mulitple PKC-independent pathways are activated in intrinsic resistance model, JIMT-1

**Figure 2:**
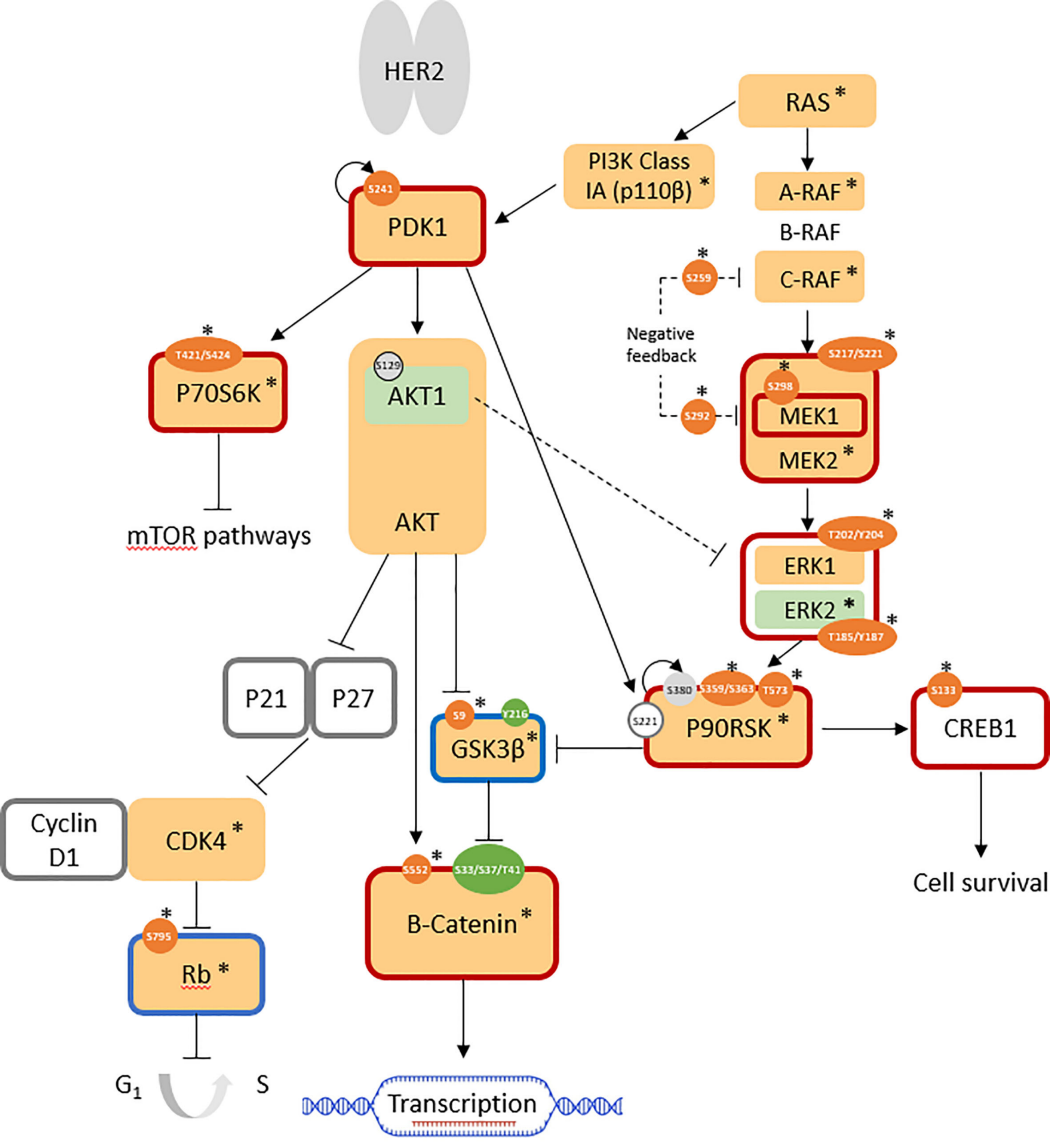
Pathways involved in JIMT-1 trastuzumab resistance (log_2_ FC normalized to SKBR3 signals). AFISimilarly expressed protein: light grey; antibody not available/poor performance: no fill; overexpressed total protein ≥ 1.5-fold (log_2_ FC ≥ 0.58): light orange; ≥ 2-fold: *; under-expressed total protein ≤0.67 (log_2_ FC ≤ -0.58): light green; ≤ 0.5-fold: *; overexpressed phosphosite ≥ 1.5-fold: orange; ≥ 2-fold: *; underexpressed phosphosite ≤ 0.67: green; ≤ 0.5-fold: *; predicted active protein: dark red outline; predicted inactive protein: dark blue outline.

Upon phosphoprotein profiling of JIMT-1 as a HER2-positive breast cancer cell line with intrinsic trastuzumab resistance, it was immediately evident that multiple cell survival and proliferation pathways were simultaneously upregulated in comparison with SKBR3, but these did not involve PKC proteins (**Figure 2**). Specifically, the RAS/RAF/MEK/ERK pathway was highly activated, together with the overexpression of the highly important kinases, PI3K class Ia (p110β isoform; log_2_ FC = +1.7) and PDK1 (log_2_ FC = +0.8). Upregulated cell cycle progression was indicated by the highly over-expressed CDK4 (log_2_ FC: +3.3) and the overall downregulation of the retinoblastoma-associated protein (Rb) tumor suppressor (log_2_ FC(Rb) = +1.6; log_2_ FC(pRb^ser807/811^ = +2.2; normalized AFI(pRb^ser795^) = 189 (not detected in SKBR3)). GSK3β activity was suppressed (log_2_ FC(GSK3β) = +1.6; AFI(pGSK3β^ser9^) = 511 (not detected in SKBR3)), leading to increased expression (log_2_ FC: +7.0) and activity (non-phospho-ser33/37/thr41: AFI = 3541; not detected in SKBR3) of β-catenin, which is associated with an increase in transcriptional activation. Enhanced cell survival was indicated by the overall activation of the cAMP-response-element-binding protein (CREB); despite total protein expression being below the cutoff in all cell lines, the active phosphosite at ser133 was not expressed in SKBR3 but expressed (normalized AFI = 177) in JIMT-1 (**Figure 2**; **Table 1**).

Control of these complex signal transduction cascades by feedback loop mechanisms makes the interpretation of some phospho-proteomic results more challenging. Specifically, both activators of the S6 ribosomal protein (RPS6), the p70S6 kinase (p70S6K/S6K1) and the ribosomal S6 kinase (p90RSK/RSK1), were activated in both models of resistance (i.e. T15 and JIMT-1), while RPS6 itself was downregulated in both cell lines. Activation of p70S6K was confirmed by the over-representation of its phosphorylation target on mTOR at serine 2448 (47), while activation of RSK1 was confirmed by the over-representation of different activating phosphosites, in both models (**Figures 1**, **2**: **Table 1**). Also of interest, deregulation of PP2A and the HER2-MYC-PP2A axis were not apparently involved in the intrinsic resistance of JIMT-1 to trastuzumab or the resistance acquired by T15. The PP2A C regulatory subunit was overexpressed at a log_2_ FC of 0.83 in JIMT-1 and was not significantly differentially expressed in T15, while its inactivating phosphosite p.tyr307 was significantly underexpressed in both cell lines. Meanwhile, no change in expression of c-myc was observed in the two cell lines in relation to SKBR3 (**Table 1**).

## Clinical perspectives

In this study, we focused on the differential protein expression and phosphorylation events in a cellular model of intrinsic resistance (JIMT-1) and one with generated trastuzumab-induced acquired resistance (T15). PKC-mediated MEK/ERK pathway activation was observed in the acquired model (T15) only. Apart from its above-mentioned functions, PKC-α expression maintains the invasiveness of triple-negative breast cancer (TNBC) and endocrine resistant cell lines through upregulation of FOXC2, a transcriptional repressor of p120-catenin (CTNND1); a high FOXC2:CTNND1ratio was also associated with shorter disease free survival in TNBC patients in The Cancer Genome Atlas (TCGA) dataset (48). FOXC2 is an epithelial-mesenchymal transition (EMT) marker, a process known to be significantly associated with HER2-positive, metastatic breast cancer in the clinical setting (49). Cells undergoing EMT commonly show upregulation of metalloproteinases (50, 51), which promote HER2 cleavage/shedding and thus a high ratio of p95:p185 HER2, associated with trastuzumab resistance and poor disease-free survival in HER2+ breast cancer (52). Assessment of the p95:p185 HER2 ratio in plasma exosomes derived from HER2-positive breast cancer patients (30) is a potential tool for the detection of early metastatic disease and monitoring of response to trastuzumab therapy.

Using the DigiWest^®^ methodology, we interrogated major signal transduction pathways to understand the complex interplay of these pathways and changes following resistance to therapy. Bead-based, multiplex (phospho)protein assays are a very efficient means of studying these pathways, whereby the supporting data from many members of the same pathway, rather than a few candidates (as is permitted by traditional Western blotting techniques) lends robustness to the overall observations. The use of this methodology to characterise exosomes for HER2 receptor ratios, FOXC2 and other EMT markers, metalloproteases, TGFβ; and PD-L1, and other markers of therapeutic resistance can accompany the other developments in liquid biopsy, such as circulating tumour cells (CTCs) (53) and patterns of cell-free nucleic acids in plasma (54), as well as protein biomarkers in other biofluids such as tears (55), to predict disease development and progression. The potential use of DigiWest^®^ to quantitate proteins from various sources provides a multiplex method that can be translated to the clinic, since ultra-high throughput proteomics by mass spectroscopy remain challenging to use in the clinical setting. Understanding treatment resistance mechanisms and incorporating multiplex assays in personalised medicine allows the prediction of early therapeutic resistance and prevents the use of non-beneficial therapies.

## Conclusion

MAPK (ERK) pathway activation was common to both intrinsic and acquired resistance cellular models. PKC-mediated MEK/ERK pathway activation in the cellular model of acquired trastuzumab resistance generated in this study (T15) was not observed in the intrinsic model, JIMT-1, which is in turn characterized by the PKC-independent activation of various pathways, including PI3K/Akt and RAS/RAF/MEK/ERK activation, β catenin stabilization by inhibitory phosphorylation of GSK3β, cell cycle progression by Rb suppression, and CREB-mediated cell survival. This is a novel observation which merits further investigation that can lead to new therapeutic combinations in HER2-poitive breast cancer with acquired therapeutic resistance to trastuzumab.

## Data availability statement

The original contributions presented in the study are included in the article/supplementary material. Further inquiries can be directed to the corresponding author.

## Author contributions

JS carried out the experiments and data analysis and contributed to the draft of the manuscript. FS-R and SF supervised JS during DigiWest analysis that was performed at the NMI institute under the approval of MT. The data analysis was performed using tools provided by MT. CS and GG conceived the study, designed and coordinated the project. GG contributed to the writing of the manuscript. All authors contributed to the article and approved the submitted version.

## Funding

This study was funded by the ALIVE Charity Foundation through the Research Innovation and Development Trust (RIDT), providing a scholarship to JS and bench fees. This work received financial support from the State Ministry of Baden-Wuerttemberg for Economic Affairs, Labour and Tourism.

## Acknowledgments

We would like to acknowledge the Institute of Molecular Medicine & Biobanking and the Faculty of Medicine& Surgery for the support in the use of the facilities at the University of Malta.

## Conflict of interest

The authors declare that the research was conducted in the absence of any commercial or financial relationships that could be construed as a potential conflict of interest.

## Publisher’s note

All claims expressed in this article are solely those of the authors and do not necessarily represent those of their affiliated organizations, or those of the publisher, the editors and the reviewers. Any product that may be evaluated in this article, or claim that may be made by its manufacturer, is not guaranteed or endorsed by the publisher.

## References

[1] Patani N Martin LA Dowsett M. Biomarkers for the clinical management of breast cancer: International perspective. Int J Cancer (2013) 133:1–13. doi: 10.1002/ijc.27997 23280579

[2] Graus-Porta D Beerli RR Daly JM Hynes NE. ErbB-2, the preferred heterodimerization partner of all ErbB receptors, is a mediator of lateral signaling. EMBO J (1997) 16:1647–55. doi: 10.1093/emboj/16.7.1647 PMC1169769 9130710

[3] Bose R Molina H Patterson AS Bitok JK Periaswamy B Bader JS. Phosphoproteomic analysis of Her2/neu signaling and inhibition. Proc Natl Acad Sci U.S.A. (2006) 103:9773–8. doi: 10.1073/pnas.0603948103 PMC1502529 16785428

[4] Wong LL Chang CF Koay ES Zhang D. Tyrosine phosphorylation of PP2A is regulated by HER-2 signalling and correlates with breast cancer progression. Int J Oncol (2009) 34:1291–301. doi: 10.3892/ijo_00000256 19360341

[5] Janghorban M Farrell AS Allen-Petersen BL Pelz C Daniel CJ Oddo J. Targeting c-MYC by antagonizing PP2A inhibitors in breast cancer. Proc Natl Acad Sci U.S.A. (2014) 111:9157–62. doi: 10.1073/pnas.1317630111 PMC4078832 24927563

[6] Risom T Wang X Liang J Zhang X Pelz C Campbell LG. Deregulating MYC in a model of HER2+ breast cancer mimics human intertumoral heterogeneity. J Clin Invest (2020) 130:231–46. doi: 10.1172/JCI126390 PMC6934197 31763993

[7] Dueck AC Reinholz MM Geiger XJ Tenner K Ballman K Jenkins RB. Impact of c-MYC protein expression on outcome of patients with early-stage HER2+ breast cancer treated with adjuvant trastuzumab NCCTG (Alliance) N9831. Clin Cancer Res (2013) 19:5798–807. doi: 10.1158/1078-0432.CCR-13-0558 PMC3805021 23965903

[8] Schade B Lesurf R Sanguin-Gendreau V Bui T Deblois G O’Toole SA. β-catenin signaling is a critical event in ErbB2-mediated mammary tumor progression. Cancer Res (2013) 73:4474–87. doi: 10.1158/0008-5472.CAN-12-3925 23720052

[9] Hudis CA. Trastuzumab – mechanism of action and use in clinical practice. N Engl J Med (2007) 357:39–51. doi: 10.1056/NEJMra043186 17611206

[10] Molina MA Codony-Servat J Albanell J Rojo F Arribas J Baselga J. Trastuzumab (herceptin), a humanized anti-Her2 receptor monoclonal antibody, inhibits basal and activated Her2 ectodomain cleavage in breast cancer cells. Cancer Res (2001) 61:4744–9. 11406546

[11] Yakes FM Chinratanalab W Ritter CA King W Seelig S Arteaga CL. Herceptin-induced inhibition of phosphatidylinositol-3 kinase and akt is required for antibody-mediated effects on p27, cyclin D1, and antitumor action. Cancer Res (2002) 62:4132–41. 12124352

[12] Klapper LN Waterman H Sela M Yarden Y. Tumor-inhibitory antibodies to HER-2/ErbB-2 may act by recruiting c-cbl and enhancing ubiquitination of HER-2. Cancer Res (2000) 60:3384–8. 10910043

[13] Yen L You XL Al Moustafa AE Batist G Hynes NE Mader S. Heregulin selectively upregulates vascular endothelial growth factor secretion in cancer cells and stimulates angiogenesis. Oncogene (2000) 19:3460–9. doi: 10.1038/sj.onc.1203685 10918604

[14] Clynes RA Towers TL Presta LG Ravetch JV. Inhibitory fc receptors modulate *in vivo* cytoxicity against tumor targets. Nat Med (2000) 6:443–6. doi: 10.1038/74704 10742152

[15] Pietras RJ Fendly BM Chazin VR Pegram MD Howell SB Slamon DJ. Antibody to HER-2/neu receptor blocks DNA repair after cisplatin in human breast and ovarian cancer cells. Oncogene (1994) 9:1829–38. 7911565

[16] Nahta R Yu D Hung MC Hortobagyi GN Esteva FJ. Mechanisms of disease: understanding resistance to HER2-targeted therapy in human breast cancer. Nat Clin Pract Oncol (2006) 3:269–80. doi: 10.1038/ncponc0509 16683005

[17] Price-Schiavi SA Jepson S Li P Arango M Rudland PS Yee L. Rat Muc4 (sialomucin complex) reduces binding of anti-ErbB2 antibodies to tumor cell surfaces, a potential mechanism for herceptin resistance. Int J Cancer (2002) 99:783–91. doi: 10.1002/ijc.10410 12115478

[18] Nagy P Friedländer E Tanner M Kapanen AI Carraway KL Isola J. Decreased accessibility and lack of activation of ErbB2 in JIMT-1, a herceptin-resistant, MUC4-expressing breast cancer cell line. Cancer Res (2005) 65:473–82. doi: 10.1158/0008-5472.473.65.2 15695389

[19] Seo AN Lee HJ Kim EJ Jang MH Kim YJ Kim JH. Expression of breast cancer stem cell markers as predictors of prognosis and response to trastuzumab in HER2-positive breast cancer. Br J Cancer (2016) 114:1109–16. doi: 10.1038/bjc.2016.101 PMC4865964 27115469

[20] Molina MA Sáez R Ramsey EE Garcia-Barchino MJ Rojo F Evans AJ. NH(2)-terminal truncated HER-2 protein but not full-length receptor is associated with nodal metastasis in human breast cancer. Clin Cancer Res (2002) 8:347–53. 11839648

[21] Ozkavruk Eliyatkin N Aktas S Ozgur H Ercetin P Kupelioglu A. The role of p95HER2 in trastuzumab resistance in breast cancer. J BUON (2016) 21:382–9. 27273948

[22] Nagata Y Lan KH Zhou X Tan M Esteva FJ Sahin AA. PTEN activation contributes to tumor inhibition by trastuzumab, and loss of PTEN predicts trastuzumab resistance in patients. Cancer Cell (2004) 6:117–27. doi: 10.1016/j.ccr.2004.06.022 15324695

[23] Berns K Horlings HM Hennessy BT Madiredjo M Hijmans EM Beelen K. A functional genetic approach identifies the PI3K pathway as a major determinant of trastuzumab resistance in breast cancer. Cancer Cell (2007) 12:395–402. doi: 10.1016/j.ccr.2007.08.030 17936563

[24] Lu Y Zi X Zhao Y Mascarenhas D Pollak M. Insulin-like growth factor-I receptor signaling and resistance to trastuzumab (Herceptin). J Natl Cancer Inst (2001) 93:1852–7. doi: 10.1093/jnci/93.24.1852 11752009

[25] Lu Y Zi X Pollak M. Molecular mechanisms underlying IGF-i-induced attenuation of the growth-inhibitory activity of trastuzumab (Herceptin) on SKBR3 breast cancer cells. Int J Cancer (2004) 108:334–41. doi: 10.1002/ijc.11445 14648698

[26] Shattuck DL Miller JK Carraway KL Sweeney C. Met receptor contributes to trastuzumab resistance of Her2-overexpressing breast cancer cells. Cancer Res (2008) 68:1471–7. doi: 10.1158/0008-5472.CAN-07-5962 18316611

[27] Chung YC Chang CM Wei WC Chang TW Chang KJ Chao WT. Metformin-induced caveolin-1 expression promotes T-DM1 drug efficacy in breast cancer cells. Sci Rep (2018) 8:1–9. doi: 10.1038/s41598-018-22250-8 29500444 PMC5834501

[28] Pandey JP Namboodiri AM. Genetic variants of IgG1 antibodies and FcγRIIIa receptors influence the magnitude of antibody-dependent cell-mediated cytotoxicity against prostate cancer cells. Oncoimmunology (2014) 3:e27317. doi: 10.4161/onci.27317 24701371 PMC3961482

[29] Martinez VG O’Neill S Salimu J Breslin S Clayton A Crown J. Resistance to HER2-targeted anti-cancer drugs is associated with immune evasion in cancer cells and their derived extracellular vesicles. Oncoimmunology (2017) 6:e1362530. doi: 10.1080/2162402X.2017.1362530 29209569 PMC5706614

[30] Ciravolo V Huber V Ghedini GC Venturelli E Bianchi F Campiglio M. Potential role of HER2-overexpressing exosomes in countering trastuzumab-based therapy. J Cell Physiol (2012) 227:658–67. doi: 10.1002/jcp.22773 21465472

[31] Paik S Tang G Shak S Kim C Baker J Kim W. Gene expression and benefit of chemotherapy in women with node-negative, estrogen receptor–positive breast cancer. J Clin Oncol (2006) 24:3726–34. doi: 10.1200/JCO.2005.04.7985 16720680

[32] van ‘t Veer LJ Dai H van de Vijver MJ He YD Hart AAM Mao M. Gene expression profiling predicts clinical outcome of breast cancer. Nature (2002) 415:530–6. doi: 10.1038/415530a 11823860

[33] Scerri J Baldacchino S Saliba C Scerri C Grech G. Bead-based RNA multiplex panels for biomarker detection in oncology samples. Methods (2019) 158:86–91. doi: 10.1016/j.ymeth.2018.10.008 30352255

[34] Kim BK Lee JW Park PJ Shin YS Lee WY Lee KA. The multiplex bead array approach to identifying serum biomarkers associated with breast cancer. Breast Cancer Res (2009) 11:R22. doi: 10.1186/bcr2247 19400944 PMC2688951

[35] Zazo S González-Alonso P Martín-Aparicio E Chamizo C Cristóbal I Arpí O. Generation, characterization, and maintenance of trastuzumab-resistant HER2+ breast cancer cell lines. Am J Cancer Res (2016) 6:2661–78. PMC5126281 27904779

[36] Treindl F Ruprecht B Beiter Y Schultz S Döttinger A Staebler A. A bead-based western for high-throughput cellular signal transduction analyses. Nat Commun (2016) 7:12852. doi: 10.1038/ncomms12852 27659302 PMC5036152

[37] Ahn J Lee JG Chin C In S Yang A Park HS. MSK1 functions as a transcriptional coactivator of p53 in the regulation of p21 gene expression. Exp Mol Med (2018) 50:1–12. doi: 10.1038/s12276-018-0160-8 PMC6180136 30305627

[38] Hsu YL Hou MF Kuo PL Huang YF Tsai EM. Breast tumor-associated osteoblast-derived CXCL5 increases cancer progression by ERK/MSK1/Elk-1/Snail signaling pathway. Oncogene (2013) 32:4436–47. doi: 10.1038/onc.2012.444 23045282

[39] Parker P Murray-Rust J. PKC at a glance. J Cell Sci (2004) 117:131–2. doi: 10.1242/jcs.00982 14676268

[40] Uhlén M Fagerberg L Hallström BM Lindskog C Oksvold P Mardinoglu A. Proteomics. tissue-based map of the human proteome. Science (2015) 347:1260419. doi: 10.1126/science 25613900

[41] Emmanouilidi A Falasca M. Targeting PDK1 for chemosensitization of cancer cells. Cancers (Basel) (2017) 9:140. doi: 10.3390/cancers9100140 PMC5664079 29064423

[42] Singh RK Kumar S Gautam PK Tomar MS Verma PK Singh SP. Protein kinase c-α and the regulation of diverse cell responses. Biomol Concepts (2017) 8:143–53. doi: 10.1515/bmc-2017-0005 28841566

[43] Ways DK Kukoly CA deVente J Hooker JL Bryant WO Posekany KJ. MCF-7 breast cancer cells transfected with protein kinase c-alpha exhibit altered expression of other protein kinase c isoforms and display a more aggressive neoplastic phenotype. J Clin Invest (1995) 95:1906–15. doi: 10.1172/JCI117872 PMC295735 7706498

[44] Gupta AK Galoforo SS Berns CM Martinez AA Corry PM Guan KL. Elevated levels of ERK2 in human breast carcinoma MCF-7 cells transfected with protein kinase c alpha. Cell Prolif (1996) 29:655–63. doi: 10.1111/j.1365-2184.1996.tb00979.x 9146728

[45] Bailey TA Luan H Tom E Bielecki TA Mohapatra B Ahmad G. A kinase inhibitor screen reveals protein kinase c-dependent endocytic recycling of ErbB2 in breast cancer cells. J Biol Chem (2014) 289:30443–58. doi: 10.1074/jbc.M114.608992 PMC4215227 25225290

[46] Magnifico A Albano L Campaner S Campiglio M Pilotti S Ménard S. Protein kinase c alpha determines HER2 fate in breast carcinoma cells with HER2 protein overexpression without gene amplification. Cancer Res (2007) 67:5308–17. doi: 10.1158/0008-5472.CAN-06-3936 17545611

[47] Chiang GG Abraham RT. Phosphorylation of mammalian target of rapamycin (mTOR) at ser-2448 is mediated by p70S6 kinase. J Biol Chem (2005) 280:25485–90. doi: 10.1074/jbc.M501707200 15899889

[48] Pham TND Perez White BE Zhao H Mortazavi F Tonetti DA. Protein kinase c α enhances migration of breast cancer cells through FOXC2-mediated repression of p120-catenin. BMC Cancer (2017) 17:832. doi: 10.1186/s12885-017-3827-y 29216867 PMC5719564

[49] Giordano A Gao H Anfossi S Cohen E Mego M Lee BN. Epithelial-mesenchymal transition and stem cell markers in patients with HER2-positive metastatic breast cancer. Mol Cancer Ther (2012) 11:2526–34. doi: 10.1158/1535-7163.MCT-12-0460 PMC3500676 22973057

[50] Duhachek-Muggy S Qi Y Wise R Alyahya L Li H Hodge J. Metalloprotease-disintegrin ADAM12 actively promotes the stem cell-like phenotype in claudin-low breast cancer. Mol Cancer (2017) 16:32. doi: 10.1186/s12943-017-0599-6 28148288 PMC5288940

[51] Nami B Wang Z. HER2 in breast cancer stemness: A negative feedback loop towards trastuzumab resistance. Cancers (Basel) (2017) 9:40. doi: 10.3390/cancers9050040 PMC5447950 28445439

[52] Feldinger K Generali D Kramer-Marek G Gijsen M Ng TB Wong JH. ADAM10 mediates trastuzumab resistance and is correlated with survival in HER2 positive breast cancer. Oncotarget (2014) 5:6633–46. doi: 10.18632/oncotarget.1955 PMC4196152 24952873

[53] Ignatiadis M Rothé F Chaboteaux C Durbecq V Rouas G Criscitiello C. HER2-positive circulating tumor cells in breast cancer. PloS One (2011) 6:e15624. doi: 10.1371/journal.pone.0015624 21264346 PMC3018524

[54] Crigna AT Samec M Koklesova L Liskova A Giordano FA Kubatka P. Cell-free nucleic acid patterns in disease prediction and monitoring - hype or hope? EPMA J (2020) 11:603–27. doi: 10.1007/s13167-020-00226-x PMC7594983 33144898

[55] Zhan X Li J Guo Y Golubnitschaja O. Mass spectrometry analysis of human tear fluid biomarkers specific for ocular and systemic diseases in the context of 3P medicine. EPMA J (2021) 12:449–75. doi: 10.1007/s13167-021-00265-y PMC8639411 34876936

